# Prevalence of *Trypanosoma cruzi* Infection in Pregnant Women and Risk of Vertical Transmission in Newborns in Chiapas, Mexico

**DOI:** 10.3390/tropicalmed9110261

**Published:** 2024-11-01

**Authors:** Sury Antonio López Cancino, Leticia Eligio García, María del Pilar Crisóstomo Vázquez, Mariana Soria Guerrero, Enedina Jiménez Cardoso, Marcos Meneses Mayo, Sergio Agustín Islas Andrade

**Affiliations:** 1 Head of Health Research Office, IMSS BIENESTAR State Coordination Chiapas, Health Services of the Mexican Institute of Social Security for Welfare (IMSS-BIENESTAR), 16 South West 333, Tuxtla Gutiérrez 29067, Mexico; 2 Parasitology Research Laboratory, Children Hospital of México “Federico Gómez”, Dr. Márquez 162, Col Doctores, Cuauhtémoc, Mexico City 06720, Mexico; leticiaeligio@yahoo.com.mx (L.E.G.); crisostomopilar@gmail.com (M.d.P.C.V.); mariguerrero1400@gmail.com (M.S.G.); 3 Faculty of Health Science, Anahuac Mexico University, Av. Anahuac University 46, Lomas Anáhuac, Huixquilucan, Estado de México, Huixquilucan 52786, Mexico; marcos.meneses@anahuac.mx (M.M.M.); sergio.islas@anahuac.mx (S.A.I.A.)

**Keywords:** congenital, Chagas disease, *Trypanosoma cruzi*, pregnancy, newborn

## Abstract

The Mexican state of Chiapas is considered epidemiologically significant for Chagas disease due to the coexistence of infected reservoirs and vectors, including migratory populations from Central and South America. However, there is a lack of monitoring programs for the timely detection of this disease. The objective of this study was to elucidate the prevalence of *Trypanosoma cruzi* infection in pregnant women and the risk of vertical transmission in newborns at two hospitals located in the Metropolitan Region of Tuxtla Gutierrez, the capital of Chiapas State Mexico. A cross-sectional study was carried out with 193 pregnant women with gestational ages between 32 and 40 weeks, who underwent immunological testing to diagnose Chagas disease. Conventional PCR testing on cord blood revealed the presence of *T. cruzi* in newborns. The prevalence of *T. cruzi* infection in pregnant women was 32.12% (95% confidence interval (CI): 0.25, 0.38). The 62 pregnant women who tested positive for Chagas disease gave birth to 63 children, and in 5 newborns (8% (5/62), 95% confidence interval (CI): 0.02, 0.19), PCR tests on umbilical cord blood were positive for *T. cruzi*. In conclusion, the dataset showed a high prevalence of Chagas disease in the sample of pregnant women studied and a maternal–fetal transmission rate of 8%.

## 1. Introduction

Chagas disease (CD), caused by *Trypanosoma cruzi*, is classified as a neglected tropical disease transmitted by triatomine bugs (Family Reduviidae, subfamily Triatominae) in Latin America [1]. Due to migratory flows, congenital Chagas disease transmission is gaining increasing importance, particularly in Europe and the USA, where the disease has become a factor of apprehension for public health [2,3]. Congenital Chagas disease is considered an acute parasitic infection. Before giving birth, infected mothers may experience premature rupture of membranes, poor fetal growth, miscarriage, or stillbirths [4,5].

Congenitally infected newborns are typically asymptomatic at birth, almost always showing prematurity and preterm birth, low birthweight, and low Apgar scores. In the worst cases, the symptomatic infected babies present respiratory distress syndrome, hepatosplenomegaly, anemia, thrombocytopenia, and even central nervous system lesions [6]. These signs can be attributed to the parasite load, the parasite’s DTU, and the baby’s immune response. Some cases may be clinically severe and lead to death. Cases not detected due to a lack of symptoms may lead to the development of gastrointestinal and cardiac disease in the future, as well as increase the risk of disability if it is not diagnosed [7,8,9].

The first case of congenital Chagas disease (CCd) in Mexico was recorded in a community from Oaxaca in 1998 [10]. However, only 7 studies have been carried out from 2006 to 2018 in at least 9 states of the country, showing a variable prevalence of between 1% and 12% (using a global sample of 4616). Four of these studies implemented diagnostic tests for *T. cruzi* infection in newborns (Supplementary Table S1) [11,12,13,14,15,16]. Only 1 study carried out in 2 communities in the state of Chiapas, located near the border with Guatemala, reported a prevalence of Chagas disease of 2.04% (in a sample of 1125 pregnant women), and a frequency of maternal–fetal transmission of 22.2% (2/9) in Tapachula and 7.14% (1/14) in Palenque [12]. Recently, a study carried out at the Hospital General de Mexico in Mexico City estimated the prevalence of Chagas disease at 20% in 150 screened patients [15]. However, there is no Chagas disease screening program for women who are pregnant or of childbearing age in Mexico.

The diagnosis of *T. cruzi* infection by immunological tests during pregnancy and the microscopic or molecular testing in the newborns represents an opportunity for early detection of the parasite, assisting in effectively treating the parasite [1,17,18,19,20]. Regrettably, so far there is no Chagas disease screening program for pregnant women or those at childbearing age in Mexico. Therefore, the aim of this study was to determine the prevalence of *T. cruzi* infection in pregnant women and the risk of vertical transmission in newborns in two hospitals in the metropolitan region of Tuxtla Gutierrez, capital of the state of Chiapas (southern Mexico).

## 2. Materials and Methods

### 2.1. Study Setting

This study was performed in the metropolitan zone of Tuxtla Gutierrez, in the central region of Chiapas. The metropolitan zone includes urban-to-rural communities positioned at different altitudes from 175 to 1763 m above sea level. The climate is warm and sub-humid, with an average annual rainfall of 915 mm, the majority of which falls in the summer. The city of Tuxtla Gutierrez is the main economic center of the state. The population of the metropolitan zone is 848,274 inhabitants [21]. Chronic Chagas disease cases, as well as infected *Triatoma dimidiata* bugs and mammals, have been reported with variable infection rates for suburban and rural areas of the metropolitan area and are considered a pathway to migration from rural communities, including both national and foreign populations [22].

### 2.2. Patient Cohorts, Sample Collections, and Maternal Obstetrical Histories

In 2019, we conducted a cross-sectional study, enrolling 193 women ranging from 32 to 40 weeks of pregnancy without a previous diagnosis of Chagas disease. The included participants were attended to at two hospitals located in Chiapas: 174 in the Regional Hospital (RH) of the Ministry of Health in the city of Tuxtla Gutierrez, and 19 in the Medical Unit of Specialties (MUS) in the city of Chiapa de Corzo.

The ethical considerations followed the guidelines set by the Ethical Institutional Committee of the Children’s Hospital of Mexico (under Dr. Federico Gómez), which reviewed and approved the project (HIM-2017-063). Written and informed consent was obtained from all participants before delivery, signed either by themselves or by a responsible family member, according to each case.

All participants were interviewed to collect information about their physical, obstetric, clinical, and epidemiological features. All provided answers were recorded for further analysis. After delivering, a neonatologist physician conducted a medical examination of each newborn, confirming the sex, determining gestational age testing, estimating the Apgar score, measuring birthweight, size, and head circumference, and identifying abnormalities in the liver, spleen, heart, and digestive tract.

For each participant, a 10 mL blood sample was obtained from a venous puncture in the brachial vein prior to normal delivery or cesarean section. A measure of 5 mL was placed into assay tubes containing an anticoagulant (EDTA), while the other 5 mL was placed into anticoagulant-free assay tubes. After delivery, a blood sample was obtained from the umbilical cord via puncture. When this was not possible, a blood sample was taken from the placenta. A measure of 0.5 µL of whole blood was taken for screening using an immunochromatographic Chagas Stat-Pak^®^ test (Chembio Diagnostic System, Medford, NY, USA) and performed promptly for all participants, following the manufacturer’s protocol. The results were obtained within 15 min [18,23]. Here, we corroborated the outcomes of the immunochromatographic Chagas Stat-Pak^®^ test, using the same batch to evidence the reactivity found in situ. We used sera from family members from Chiapas, which proved positive for Chagas disease.

After collecting the blood, all samples were allowed to clot by leaving them undisturbed at room temperature for 30 min. Clots were removed using centrifugation at 2000× *g* for 10 min, and the resulting sera were removed using a micropipette. The serum samples were aliquoted, labeled, and stored at −20 °C until use for ELISA Chagas testing. The samples with the anticoagulant (EDTA) were processed for genomic DNA extraction using a GeneJET™ Kit (Genomic DNA Purification Kit, Thermo Fisher Scientific™, Waltham, MA, USA), following the manufacturer’s instructions. The DNA pellet was resuspended in nuclease-free water and preserved at −20 °C for subsequent molecular testing with conventional PCR in the Parasitology Research Laboratory of the Children’s Hospital of Mexico (under Dr. Federico Gómez).

### 2.3. Maternal Serological Diagnosis of Chagas Disease

An indirect immunoenzymatic ELISA (homemade ELISA) with *T. cruzi* total antigen was performed and previously validated using 96-well polystyrene plates and sensitized with 5 μg/mL of total *T. cruzi* antigen (CL Brener) [24,25]. The sera to be analyzed were diluted at 1:250 in PBS:5% milk, and the conjugate anti-human IgG coupled to peroxidase was used at a dilution of 1:5000. The reaction was developed with hydrogen peroxide and ortho-phenylenediamine as a substrate, and incubation was carried out at room temperature until a colorful reaction was observed. The enzymatic reaction was stopped by adding 100 μL of 4M sulfuric acid. The color intensity of the reaction was determined photometrically at 450 nm with an ELISA reader (Stat Fax 4200 Awareness Technology Inc, Palm City, FL, USA). Based on the third-generation indirect method due to the use of recombinant antigens for the detection of antibodies against *T. cruzi* in human sera or plasma samples, a recombinant micro-ELISA assay (ACCUTRACK CHAGAS ELISA^®^ Laboratorio Lemos S.R.L. Santiago del Estero, Buenos Aires, Argentina) with a sensitivity of 100% and a specificity of 99% was performed following the manufacturer’s instructions [25,26,27].

The cut-off values for both ELISA assays (homemade and Accutrack Chagas recombinant ELISA) were estimated using 26 serum samples obtained from people without Chagas (mean + 2 SD) as the control. Positive absorbance was considered at 0.453 ± 2. The gray zone ranged from 0.431 to 0.475 optical density.

Both tests were administered to all participants, who were considered positive only when both tests were positive. Discrepancies between both ELISA assays were solved by repeating the tests.

### 2.4. T. cruzi PCR Detection in Umbilical Cord Blood

To detect *T. cruzi* DNA, the nuclear primers Tcz1 (5′-CGA-GCT-CTT-GCC-CAC-ACG-GGT-GCT-3′) and Tcz2 (5′-CCT-CCA-AGC-AGC-GGA-TAG-TTC-AGG-3′) were used and amplified with a conventional polymerase chain reaction (PCR) [28].

To amplify *T. cruzi* DNA, the PCR reaction mixture contained a 1X buffer solution (10 mM Tris-HCl; 50 mM), 1.5 mM of Cl2Mg, 0.2 mM of dNTPs (dATP, dCTP, dGTP, and dTTP), 50 nM of primers, 300 ng of patient gDNA, and 0.12 U of Taq DNA polymerase (Ampli Taq Gold™ 360 Master Mix, Thermo Fisher Scientific Inc.), for a final volume of 25 μL. The assay was developed following an initial denaturation step at 94 °C for 5 min, followed by 40 cycles at 94 °C for 20 s, 57 °C for 10 s, and 72 °C for 30 s, with a terminal extension at 72 °C for 7 min in a Techne^®^ thermocycler (Thermo Fisher Scientific, Inc.).

The PCR products were loaded on 1% agarose gel and stained with ethidium bromide. Electrophoresis was performed at 80 mV for 45 min, after which the gel was visualized under UV light and photographed. For all experiments, DNA from the CL Brener *T. cruzi* strain and DNA from a patient with Chagas were used as positive controls.

### 2.5. Statistical Analysis

Information analysis was conducted using Minitab 18 software. Data were evaluated using the chi-square test. We used Fisher’s exact test to estimate whether there were differences among the hospital prevalences. For both tests, *p*-values ≤ 0.05 were considered statistically significant, considering a confidence interval of 95%.

## 3. Results

### 3.1. Description of the Sample

In this study, 193 women and their newborns were included. Of the patients who attended the Regional Hospital, the majority (90%) came from the metropolitan zone of Tuxtla Gutiérrez (54.6% from Tuxtla Gutiérrez, 8.6% from Chiapa de Corzo, 2.3% from Berriozábal, 5.7% from Suchiapa, and 7.5% from San Fernando), while 2.9% came from Villaflores and Ixtapa, and the remaining patients came from municipalities ranging from 100 to 200 km away (15.5%). Two patients were from Oaxaca and Tabasco, Mexico (1.7%). Of the patients who attended the MUS, the majority (74%) came from Chiapa de Corzo, Tuxtla Gutiérrez, and localities around the metropolitan zone of Tuxtla Gutiérrez (Supplementary Information Table S2). The mean age was 25.7 (SD 5), and there were no statistically significant differences between hospitals (*p* < 0.05).

### 3.2. Gynecological History

The gestational ages are shown in Table 1. The frequency of previous pregnancies was rated from one to seven. One 40-year-old participant had 12 viable deliveries. In this study, 66% (127) of participants gave birth via normal deliveries, while 34% (66) underwent cesarean sections. A total of 1.4% had twins, while the same proportion (1.4%) experienced stillbirths. Complications during or after normal delivery or cesarean section were not registered. Other gynecological and obstetric features are shown in Table 1.

### 3.3. Clinical Characteristics of Neonates

In this study, the sample size of newborns was 195 due to the inclusion of 2 pairs of twin deliveries. The sex of neonates was predominantly male, with boys comprising 51% and girls comprising 49%. The majority (98%) of newborns had a normal birthweight for gestational age (AGA), with an average of 3109.22 g (SD 404, range 1860–4050). The remaining 2% had an abnormal birthweight for gestational age, with an average of 570 g (SD 98.9, range 500–640). All newborns were compatible with life and showed no symptoms of congenital Chagas disease, including newborns with non-AGA. The average age estimated by CAPURRO was 38.7 weeks. The two pairs of twins (1.4%) were born between 26 and 38 weeks, and all required medical assistance in the Intensive Care Unit. Stillbirths occurred between 26 and 35 weeks. The clinical characteristics of the newborns are described in Table 2. All newborns who tested positive were asymptomatic. The Apgar score was 8 at one minute and 9 at five minutes. Morphometric data on newborns who tested positive, such as birthweight and size, were found to be within a normal range.

### 3.4. Prevalence of T. cruzi Infection in Pregnant Women and Newborns

Overall, 193 pregnant women tested with immunochromatographic Chagas Stat-Pak^®^ had negative results. Despite the discrepancies, serological tests revealed that 32.12% (62/193) were positive in both ELISA assays (95% confidence interval (CI): 0.25, 0.38). Non-significant differences were found between groups of participants based on hospitals (Fisher’s exact test, *p* < 0.05; Table 3). Interestingly, a participant with a twin pregnancy tested positive for *T. cruzi* infection. The PCR test for *T. cruzi* infection through cord blood of positive participants was 8% (5/62; 95% confidence interval (CI): 0.066, 0.234).

### 3.5. Distribution of Prevalences and Risk Factors

The geographic distribution of *T. cruzi* prevalence was higher in metropolitan municipalities, with 50% located in Tuxtla Gutiérrez, 10% in Chiapa de Corzo, 10% in San Fernando, 8% in Suchiapa, and the remaining 21% in surrounding areas (Supplementary Table S2). Among the newborns, 60% (3/5) resided within the metropolitan area, while the rest (40%) were from the Mezcalapa and Frailesca regions.

Risk factors detected in all participants who tested positive for *T. cruzi* infection included 9.6% (6/62) who reported an awareness of the triatomine vector, while only 3.2% (2/62) indicated having had contact with the insect at any time (odds ratio: 1.42, Chi2: 0.14; *p* > 0.05). Regarding travel history, only one of the positive participants reported having traveled to Cancun (Quintana Roo) and Mexico City, one month and one year before sampling. Additionally, 6.4% of participants who were positive were born in Mexico City, Oaxaca, Veracruz, or Coahuila. Other risk factors are shown in Table 4.

## 4. Discussion

The main center for gynecological and obstetric services in the metropolitan zone of Tuxtla Gutiérrez, Chiapas, is the Regional Hospital “Dr. Rafael Pascacio Gamboa”, principally for residents and patients referred for obstetric risk or other pregnancy complications from different municipalities or states. However, there is no surveillance system for Chagas disease in women or newborn babies, despite the implementation of initiatives, such as the elimination of mother-to-child transmission (EMTCT) initiative for PAHO Member States, which has been in place since 2010 [29]. In this study, we tested a group of pregnant women using two different tests. With the immunochromatic rapid test (Stat-Pak^®^), no patient samples were detected for *T. cruzi* infection, while with ELISA tests (a homemade ELISA and a commercial micro-ELISA Accutrack Chagas ELISA^®^ assay), 62 patients tested positive.

### 4.1. Prevalence of T. cruzi Infection in Pregnant Women

The discrepancies related to the *T. cruzi* infection rates in mothers are possibly due to the Chagas disease phase and parasitemia level, which influences the immune response. Additionally, they may also be related to the sensitivity of the rapid tests used, the specific response to the DTU, and the ample antigenic diversity of *T. cruzi* circulating in the area. In Mexico, the most predominant DTU is TcI, but TcIV has also been detected [17,19,29]. Therefore, further implementing the two different serological methods (homemade and commercial ELISA tests based on distinct antigenic principles) was necessary to confirm the Chagas disease cases presented herein, which is in agreement with the current diagnosis algorithm recommended by PAHO [29,30]. In this study, the first method was based on CL Brener *T. cruzi* total antigen, while micro-ELISA was based on recombinant antigens, both for the detection of antibodies against *T. cruzi* in sera but not developed with the TcI lineage. In fact, the performance of commercial serological tests with sera from Mexico is generally lower than that with sera from South America [15,19]. We hypothesize that the heterogeneous results could be related to the DTU of *T. cruzi*.

An indirect estimation of Chagas disease in pregnant women in Mexico indicated a prevalence of approximately 2.2% [31]. Studies previously carried out in Chiapas have reported prevalences ranging from 1.5% to 2.6% in two urban areas bordering Guatemala, namely, Tapachula and Palenque, Chiapas, respectively [12]. In our study, we found a prevalence of 32.12% from a group of pregnant women about to give birth in two hospitals located in the metropolitan area of Tuxtla Gutiérrez. The rate reported herein exceeded the previously noted prevalence, even for states such as Jalisco, Mexico, where the prevalence has been estimated to be 12%, using a sample almost double the size of that evaluated in this study [13]. Here, the prevalences were distributed in the main four municipalities of the metropolitan area, accounting for just over 50%, while at least eleven other municipalities each reported one positive case, which could suggest the existence of additional undetected cases.

In this study, the sample size at the MSU was small; however, the global analysis of prevalence (including the sample of RH) highlighted the current importance of screening women during pregnancy as a public health policy. In this sense, the detection of positive pregnant women leads to the early identification of CCd in their neonates and provides an opportunity to administer specific treatment to eliminate parasitemia in babies, who generally tolerate the adverse effects of existing drugs (Nifurtimox and Benznidazol) better than adults [30,32].

### 4.2. Prevalence of T. cruzi Infection and Clinical Features in Newborns

The early detection of *T. cruzi* infection in women of childbearing age represents a significant challenge to public health, especially due to the risk of transference of parasites from a pregnant woman to her fetus during pregnancy or delivery, thus causing CCd in 5% of newborns [17,18,19]. Estimates of annual deliveries by pregnant women with infections in Mexico are close to 60,000, corresponding to almost 3000 cases of CCd in babies infected via vertical transmission [31]. To our knowledge, only four studies investigating vertical transmission have been carried out in Mexico, one of them in Chiapas [12,13,16,17].

The vertical transmission of *T. cruzi* can occur during delivery, leading to acute infection with detectable parasites in newborns [29]. The development of molecular tools for diagnosing CCd could help to standardize the complex heterogeneous profile of *T. cruzi* DTUs [19,30,32]. In this study, infection in babies was confirmed through a molecular method using a conventional PCR test [19], and we found that five (8%) newborns tested positive for the parasite’s DNA in cord blood. According to the WHO recommendations, two requirements are necessary—a positive serology test in the mother and positive parasitemia in the newborn after delivery—to confirm a CCd case [1]. The results were analogous to the results found in the bordering city of Palenque, Chiapas (7.4%), but a lower rate than observed in Tapachula, Chiapas (22.2%), as well as other states, including Jalisco (11.9%) and Oaxaca (20%) [12,13]. These results could suggest the establishment of the parasite cycle in urban areas, such as Tuxtla Gutierrez, a process referred to as the urbanization of a disease, occurring through non-vectorial pathways [26].

Clinical manifestations of CCd in infants are variable and present in at least a quarter of affected newborns; hence, the majority remain asymptomatic [33,34]. Consequently, diagnosis and treatment are unlikely unless active surveillance is performed [19]. However, the lack of signs of infection can mask the illness in positive infants, leading to the development of the chronic phase, which can damage the heart or other organs. That said, the clinical manifestations of CCd at birth have been clearly described as low birthweight, prematurity, hepatomegaly, splenomegaly, and respiratory distress [20,33,35]. In our study, no infants who were positive for *T. cruzi* showed symptoms related to CCd after delivery. Only a couple of living twins born to a mother who tested positive showed an abnormal birthweight for gestational age.

### 4.3. Risk Factors

Chagas disease during pregnancy represents a transmission risk of *T. cruzi* to the fetus or newborn [34,36]. Studies on risk factors for CCd are still in the early stages in Mexico; nevertheless, this transmission pathway is epidemiologically significant due to the potential for the disease to spread from endemic to nonendemic zones where the vector is not currently distributed, such as European and Asian countries, where it is considered an emerging disease [37].

The lack of knowledge of Chagas disease was evidenced in all pregnant women included in the study. Contact with the triatomine vector poses a low risk, despite the fact that the metropolitan zone is considered an endemic area for *T. dimidiata,* a vector with significant *T. cruzi* infection rates in peri-domiciliary and intra-domiciliary habitats in both rural and peri-urban settlements [38,39,40].

Similar to our study, comparable data have demonstrated high vertical transmission rates among babies born to women with chronic Chagas disease, all from vectorial transmission-free areas [41,42,43]. In our study, 9% of positive pregnant women reported awareness of the vector but denied having had recent contact. We suspect that contact could occur during childhood, likely due to the fact that participants who tested positive came from rural and suburban areas, where *T. dimidiata* could be present. Together with blood transfusion and travel history, the risk of contact with the insect nearly doubles.

## 5. Conclusions

Our results showed a high infection rate in pregnant women but a low detection rate of the *T. cruzi* parasite in their babies at two hospitals located in the metropolitan area of Chiapas. Ensuring proactive screening of all women of childbearing age will require concerted efforts to increase provider awareness, convey accurate information to the public about CD and its risks, and improve access to and the performance of diagnostic tools. These outcomes contribute to the identification of CD as a significant public health problem in women of childbearing age.

## 6. Study Limitations

This study had several key limitations. As the study was conducted in localities of the metropolitan area of Tuxtla Gutierrez, Chiapas, this may limit the generalizability of the outcomes to other regions in southern Mexico. The sample size of patients at one of the hospitals (MSU) may have introduced bias in the results, leading to an underrepresentation of the prevalence rate, even in the comparative analysis between hospitals. Another limitation was the lack of TcI use in the ELISA test, a common DTU in previous reports for Mexico. Additionally, PCR products were not sequenced, preventing association with parasite DTUs in a deeper genetic or clinical analysis. In this study, there was no follow-up on the latter evolution, despite none of the participants who tested positive developing postpartum complications, even among women who tested positive for *T. cruzi*.

## Figures and Tables

**Table 1:** Gynecological–obstetric history of participants (sample = 193).

Feature	Average/SD (Range)	Percentage (%)	Confidence Interval
Gestational age *	38.12 ± 2.32 (26–42)	--	--
Gravidity	2.48 ± 1.5	--	--
Deliveries	--	57	0.499–0.637
Cesarean	--	32	0.259–0.390
Abortion **	--	9	0.055–0.136
Stillbirth **	--	2	0.002–0.037
Multiparous	--	66	0.588–0.721
Primiparous	--	34	0.278–0.411

* During the study. ** Causes were not known. -- Does not apply.

**Table 2:** Clinical characterization of newborns (sample = 195).

Variable	Average/SD^»^/Range	Percentage (95%CI)
Newborn alive	--	98.5 (0.955–0.994)
Stillbirth	--	1.5 (0.005–0.044)
Female	--	49 (0.418–0.556)
Male	--	51 (0.443–0582)
Age (CAPURRO)	38.75 ± 2.3 (16)	--
AGA *	3109.22 g ± 404 (1860–4050)	98 (0.948–0.992)
Non-AGA	570 g ± 98.9 (500–640)	2 (0.008–0.051)

Note: All newborns were without symptoms related to congenital Chagas disease, including babies considered to have an abnormal birthweight for gestational age. SD^»^—standard deviation; * AGA—normal birthweight for gestational age.

**Table 3:** Prevalence of *T. cruzi* infection in pregnant women and newborns based on hospitals.

	RH (Positive)/95% CI	MSU (Positive)/95% CI	*p*-Value
Chagas StatPak^®^	Negative	Negative	-
ELISA ^&^	32.1% (56)/0.25–0.39	31.5% (6)/0.15–0.53	0.99 *
PCR (Newborn) ^#^	8.9% (5)/0.3–0.19	0	-

^&^ Sample sizes for pregnant women tested using ELISA: 174 in RH and 19 in MSU. ^#^ Sample size for newborns tested using PCR in RH was 56. * Fisher’s exact test.

**Table 4:** Risk factors evaluated in the group of 62 pregnant women who tested positive for *T. cruzi* infection.

Risk Factors	Percentage (%)	Absolute Frequency	Odds Ratio	Confidence Interval (95%)
Insect knowledge	9.6	6	4.57	1.10–18.93
Contact with the insect	3.2	2	1.42	0.23–8.73
History of blood transfusion	3.2	2	1.42	0.23–8.73
Travel history	3.2	2	1.42	0.23–8.73

## Data Availability

All datasets are available upon request to the corresponding author.

## Supplementary Materials

The following supporting information can be downloaded at: https://www.mdpi.com/article/10.3390/tropicalmed9110261/s1. Table S1: Studies carried out in Mexico on Chagas disease in pregnant women and the vertical transmission of *T. cruzi*. Table S2: The distribution of Chagas disease prevalence among women involved in this study from metropolitan municipalities.
