# Supplementary material for: Prevalence of Trypanosoma cruzi Infection in Pregnant Women and Risk of Vertical Transmission in Newborns in Chiapas, Mexico
Source: Trop Med Infect Dis. 2024 Nov 1;9(11):261. doi: 10.3390/tropicalmed9110261 (PMC11598654; doi:10.3390/tropicalmed9110261)
Supplement: Supplementary file 1 [file tropicalmed-09-00261-s001.zip › tropicalmed-3246404-supplementary.pdf]

Supplementary Table S1. Studies carried out in Mexico on Chagas disease in pregnant women and the vertical transmission of *T. cruzi*

**Author**	**Year**	**County/State**	**Sample size**	**Prevalence in pregnant women (%)**	**Rate of infection in newborns (%)**
Guzman-Bracho *et al.,* [10]	1998	Oaxaca	1	-	First case of congenital Chagas disease notified.
Olivera *et al.* [11]	2006	Palenque / Chiapas	60	5	WD
		Poza Rica / Veracruz	85	3.5	WD
Gamboa-León *et al.,* [15]	2011	Celaya / Guanajuato	488	0.4	WD
		Mérida / Yucatán	500	0.6	WD
*Jimenez-*Cardoso *et al.,* [13]	2012	Oaxaca	794	4.4	20
		Jalisco	557	12.02	11.9
		CDMX	97	4.12	WD
Gamboa-León *et al.,* [15]	2014	Sudzal / Yucatán	160	4.4	WD
		Teya / Yucatán	230	0.9	WD
Montes-Rincón *et al.,* [16]	2016	León / Guanajuato	520	4	0.8
Campos-Valdez *et al.,* [12]	2016	Tapachula / Chiapas	600	1.5	22.2
		Palenque / Chiapas	525	2.6	7.4
Buekens *et al.,* [14]	2018	Mérida / Valladolid Yucatán*	109	29.4	6.3
Chakravarti *et al.,* [9]	2022	Mexico City**	150	30	WD

WD: Without diagnosis, * Patients enrolled during 2011–2013 ** Patients recruited in a third level hospital. Source: own elaboration.

Supplementary Table S2. The distribution of Chagas disease prevalence among women involved in this study from metropolitan municipalities.

**Municipality**	**RH ***	**MSU***	**Prevalence**	**CI**
Tuxtla Gutiérrez*	29	2	50 % (31/62)	37.92 - 62.08
Chiapa de Corzo*	4	2	10% (6/62)	4.17 - 19.89
San Fernando	6	-	10% (6/62)	4.17 - 19.89
Suchiapa*	5	-	8% (5/62)	3.10 - 17.92
Ocozocoautla	1	1	3.2% (2/62)	0.23 - 11.67
Usumacinta	2	-	3.2% (2/62)	0.23 - 11.67
Berriozábal*	1	-	1.6% (1/62)	0.0 -9.40
Acala	-	1	1.6% (1/62)	0.0 -9.40
Copainalá	1	-	1.6% (1/62)	0.0 -9.40
Ocotepec	1	-	1.6% (1/62)	0.0 -9.40
La Concordia	1	-	1.6% (1/62)	0.0 -9.40
Francisco León	1	-	1.6% (1/62)	0.0 -9.40
Soyaló	1	-	1.6 % (1/62)	0.0 -9.40
Tapalapa	1	-	1.6 % (1/62)	0.0 -9.40
Villaflores	1	-	1.6 % (1/62)	0.0 -9.40
Oaxaca**	1	-	1.6 % (1/62)	0.0 -9.40

* Frequency of pregnant women positive for *T. cruzi* infection for each hospital.

** A southern Mexican state close Chiapas known for its significant prevalence of Chagas disease.
